# Supplementary material for: The struggle for medicine: A valid and reliable cross-sectional study on the impact of war on healthcare access and its consequences for displaced Sudanese citizens
Source: PLOS Glob Public Health. 2025 Nov 26;5(11):e0004867. doi: 10.1371/journal.pgph.0004867 (PMC12654929; doi:10.1371/journal.pgph.0004867)
Supplement: Attachment — Submitted filename: Author response.pdf [file pgph.0004867.s001.pdf]

PLOS Global Public Health

Journal Requirements:

1. Please insert an Ethics Statement at the beginning of your Methods section, under a subheading 'Ethics Statement'.
Author reply: Done

2. We note that your Data Availability Statement is currently as follows: “All relevant data are within the paper.”

Author reply: Done

Please confirm at this time whether or not your submission contains all raw data required to replicate the results of your study. Authors must share the “minimal data set” for their submission. PLOS defines the minimal data set to consist of the data required to replicate all study findings reported in the article, as well as related metadata and methods (https://journals.plos.org/plosone/s/data-availability#loc-minimal-data-set-definition).

For example, authors should submit the following data:

- The values behind the means, standard deviations and other measures reported;
- The values used to build graphs;
- The points extracted from images for analysis.

Authors do not need to submit their entire data set if only a portion of the data was used in the reported study.

If your submission does not contain these data, please either upload them as

Supporting Information files or deposit them to a stable, public repository and provide us with the relevant URLs, DOIs, or accession numbers. For a list of recommended repositories, please see https://journals.plos.org/plosone/s/recommended-repositories.

If there are ethical or legal restrictions on sharing a de-identified data set, please explain them in detail (e.g., data contain potentially sensitive information, data are owned by a third-party organization, etc.) and who has imposed them (e.g., an ethics committee). Please also provide contact information for a data access committee, ethics committee, or other institutional body to which data requests may be sent. If data are owned by a third party, please indicate how others may request data access.

Additional Editor Comments (if provided):

However, several major concerns must be addressed before the manuscript can be considered for publication:

1. Data Collection Context

Data collection occurred between October 4 and November 1, 2023, a period of significant insecurity in Sudan. Conducting consistent and safe data collection via online methods during this time appears ambitious. The authors should provide more detail on how participants were reached, and the feasibility and safety of data collection in areas experiencing active conflict, especially among internally displaced populations (IDPs).

Author reply:

We appreciate the reviewer’s valuable comment. The data collection was conducted by the four authors, who were each based in different cities across Sudan. To ensure a representative sample, we employed stratified random sampling, dividing the population based on demographic factors such as age, gender, and education level. Given the security situation, data collection was carried out exclusively through online platforms, which allowed us to reach participants, including some from internally displaced populations (IDPs), without exposing either participants or researchers to physical risk. This approach enabled safe, consistent, and geographically diverse data collection despite the challenges of ongoing conflict.

2. Sampling Strategy and Online Survey Bias

The feasibility of the stratified sampling method is unclear given the use of an online, self-administered survey. This approach likely excludes the most vulnerable groups (e.g., IDPs, people in rural areas without internet access, those with limited literacy), introducing selection bias and compromising representativeness. The authors should provide more information on the recruitment strategy, how stratification was operationalized, and a clear justification for using online data collection in a context where electricity and internet access may been severely compromised.

Author reply:

Given the severe security risks and ongoing conflict during the study period, online data collection was the only safe and feasible option to reach participants without putting researchers or respondents at risk. While we acknowledge that internet access was variable across regions, mobile internet services remained functional in many areas, including among some internally displaced populations who had access through mobile devices.

The stratified sampling approach was operationalized using available demographic information to ensure representation across key variables such as age, gender, education, and geographic distribution. Recruitment efforts were

extensive and utilized diverse online platforms (professional networks, institutional mailing lists, healthcare organizations, social media, and messaging apps), as well as collaborations with local organizations to help disseminate the survey link more broadly, including among displaced communities.

While we recognize the inherent limitations of online surveys in conflict settings, this methodology allowed us to collect timely and valuable data under extremely constrained circumstances. Moreover, it remains one of the few viable approaches in settings where physical data collection poses significant risks. The potential for selection bias has been acknowledged in the manuscript's limitations section.

3. Sample Size and Power

The sample size calculation based on Cochran’s formula indicated a minimum of 385 participants, yet only 300 were included. The authors should conduct a post hoc power analysis to assess whether the final sample size is adequate to detect the reported correlations or group differences.

Author reply:

While the initial sample size calculation using Cochran’s formula indicated a minimum target of 385 participants, logistical and security constraints during data collection limited enrollment to 300 participants.

A one-way ANOVA was conducted to evaluate the effects of income, employment status, and place of residence on medication accessibility with post hoc comparisons performed using Tukey’s HSD test. A p-value of less than 0.05 was considered statistically significant.

4. Sample Skewness

The sample is heavily skewed toward highly educated individuals (over 75% with university degrees), which is unlikely to reflect the broader Sudanese population,

especially during conflict. The implications of this sampling bias should be explicitly discussed in the limitations.

Author reply:

We acknowledge that the sample includes a high proportion of participants with university-level education (over 75%), which may limit the generalizability of the findings to the broader Sudanese population, particularly during the ongoing conflict. This distribution likely reflects the challenges associated with online data collection, where individuals with higher education levels are more likely to have consistent internet access and digital literacy. This was included in the limitation of the study (The sample may not include the most vulnerable populations and responses may have some bias.)

5. Terminology Use of “Post-War”

The use of the term “post-war” is misleading, as the war in Sudan is ongoing. The authors should revise this terminology and clearly contextualize the data collection period within the conflict timeline.

Author reply: Changed

6. Data Availability

Only summary statistics are presented in the manuscript. This does not meet PLOS's data availability requirements. The authors should upload a de-identified dataset as supplementary material or to a public repository. If there are legal or ethical restrictions, these should be clearly explained per PLOS’s exemption policy.

Author reply:

We thank the reviewer for this important comment.

The raw data are available in an Excel file containing the de-identified results with the corresponding variables used in the analysis. We are prepared to upload

this dataset as supplementary material or deposit it in a public repository in accordance with PLOS's data availability requirements.

7. Ethical Considerations

While the manuscript mentions ethics approval and informed consent, important details are missing. The authors should clarify how informed consent was obtained, how confidentiality and participant safety were ensured, and provide the ethics approval reference number for transparency.

Author reply:

Informed consent was obtained electronically from all participants prior to their participation in the online survey. The consent form included detailed information about the study’s purpose, voluntary participation, confidentiality, and data protection measures.

To ensure confidentiality and participant safety, all responses were anonymized, and no personally identifiable information was collected or stored. Data were securely stored with restricted access limited to the research team.

Ethical approval was obtained from the ethical committee of the department of clinical pharmacy; faculty of pharmacy, National university- Sudan under reference serial number was (FPNU-09-4/2023), and stated in the ethic statement.

8. Generalizability of Findings

Given the limitations of the sampling method, the findings cannot be generalized to the broader displaced or conflict-affected population in Sudan. The authors should qualify their conclusions accordingly and discuss the influence of digital exclusion on the study results.

Moreover, the manuscript appears to imply that difficulties accessing medications may have led to relocation. However, in the context of war and political

insecurity, relocation is far more likely driven by security threats than by barriers to pharmaceutical access. The authors should avoid overstating or misattributing causality unless relocation motives were directly measured. A clearer framing would treat healthcare access as a consequence, not a cause, of displacement.

Author reply:

We agree that due to the limitations inherent in our sampling method—particularly the use of online data collection—the findings cannot be generalized to the broader displaced or conflict-affected population in Sudan. We have revised the manuscript to more clearly qualify our conclusions accordingly and included a discussion on the impact of digital exclusion and its potential influence on the study results.

Regarding the issue of relocation, we appreciate the reviewer's clarification. We acknowledge that in the context of ongoing war and political insecurity, security threats are indeed the primary drivers of displacement. Our intention was to highlight that difficulties accessing medications may exacerbate the challenges faced by displaced populations rather than act as the initial cause of relocation. We have revised the manuscript to avoid implying causality and reframed healthcare access as a consequence of displacement rather than a cause.

9. Language and Presentation

The manuscript would benefit from English language editing to improve clarity, grammar, and overall readability. Several sentences could be more concise, and transitions between sections could be smoother.

Author reply

Some sentences were done

Reviewer #2: Review: The struggle for medicine: a valid and reliable cross-sectional study on the impact of war on Healthcare access and its consequences for displaced Sudanese citizens

Dear PLOS Global Public Health team

Thank you for asking me to review this important paper which I would suggest publishing with a few minor changes as detailed below. It details previously unpubished data in this setting.

The key results this original study presents are the output from a questionnaire posed to those living in Sudan prior to (and in some cases during) the recent conflict and the effects that have been had on access to medication, health care services. This is significant as nothing of this kind has been published during the recent war in Sudan and it may help inform policy makers and healthcare providers to improve the situation in this conflict and in others. The authors have conducted a literature search relating to this issue in other areas of conflict.

Data and methodology
The data was derived from a questionnaire with good detail on how this was achieved to gain representative answers.

Results
I have checked the results from the figures and text match and note these discrepancies:
-line 175: those remaining in Khartoum-text says 4.7% and Table 1 says 5.7%
-line 177: those with hypertension-text says 19.3%, Table 1 says 17.9%
-line 178: those with hypertension and diabetes, text says 17.9%, Table 1 says 16.4%
Author reply: All were corrected.

-line 186-can I ask where these figures are from in the table (Table 2)-sorry if I'm missing it!
Author reply: The figure was missed, now included (Figure 1)

I'd be interested to know what diseases the majority of respondents had (line 176) if they weren't chronic diseases. Can this be displayed?

Author reply: Unfortunately, this information cannot be displayed as the questionnaire categorized non-chronic conditions under a general "other" option without specifying disease types. As a result, we do not have detailed data on the specific diseases reported in this category.

A few comments:
-would a map of Sudan be useful with the areas sampled highlighted. This may show the uninformed reader the geography

Author reply:
It is not possible to accurately map the sampled areas.

References
All checked and refer appropriately to what is referenced in the text
References are accurate and many recent
In the introduction, (line 101) I think the reference quoted as "30" should be "25".
In order to keep the references continuous, line 129 should ideally be number 26 (Cochran's formula)

Suggested improvements

Line 118 “four authors were been”…:I would suggest rewording this sentence to be clearer, along the lines of “The four authors were from these four areas:”

Author reply:

Done
